# Using generative models to make probabilistic statements about hippocampal engagement in MEG

**DOI:** 10.1016/j.neuroimage.2017.01.029

**Published:** 2017-04-01

**Authors:** Sofie S. Meyer, Holly Rossiter, Matthew J. Brookes, Mark W. Woolrich, Sven Bestmann, Gareth R. Barnes

**Affiliations:** a Wellcome Trust Centre for Neuroimaging, Institute of Neurology, University College London, London WC1N3BG, UK; b Sir Peter Mansfield Magnetic Resonance Centre, School of Physics and Astronomy, University of Nottingham, Nottingham, UK; c Oxford Centre for Human Brain Activity, University of Oxford, Warneford Hospital, Oxford, UK; d Sobell Department for Motor Neuroscience and Movement Disorders, Institute of Neurology, University College London, London, UK

## Abstract

Magnetoencephalography (MEG) enables non-invasive real time characterization of brain activity. However, convincing demonstrations of signal contributions from deeper sources such as the hippocampus remain controversial and are made difficult by its depth, structural complexity and proximity to neocortex. Here, we demonstrate a method for quantifying hippocampal engagement probabilistically using simulated hippocampal activity and realistic anatomical and electromagnetic source modelling. We construct two generative models, one which supports neuronal current flow on the cortical surface, and one which supports neuronal current flow on both the cortical and hippocampal surface. Using Bayesian model comparison, we then infer which of the two models provides a more likely explanation of the dataset at hand. We also carry out a set of control experiments to rule out bias, including simulating medial temporal lobe sources to assess the risk of falsely positive results, and adding different types of displacements to the hippocampal portion of the mesh to test for anatomical specificity of the results. In addition, we test the robustness of this inference by adding co-registration error and sensor level noise. We find that the model comparison framework is sensitive to hippocampal activity when co-registration error is <3 mm and the sensor-level signal-to-noise ratio (SNR) is >−20 dB. These levels of co-registration error and SNR can now be achieved empirically using recently developed subject-specific head-casts.

## Introduction

Magnetoencephalography (MEG) is a non-invasive neuroimaging technique that measures electromagnetic brain activity with millisecond temporal resolution. In order to localise the spatial origin(s) of such activity, anatomical and electrophysiological information is used to constrain the solution space. Whilst this general framework is well-established for neocortical sources (Gross et al., 2003, Hämäläinen et al., 1993, Henson et al., 2009, Lopes da Silva, 2013, Vrba and Robinson, 2001), reconstruction of deep sources remains controversial (Hämäläinen et al., 1993, Mikuni et al., 1997, Riggs et al., 2009, Stephen et al., 2005). This is partly because the signal strength, and consequently also the spatial resolution, rapidly decreases with distance from the sensors (Hillebrand and Barnes, 2002), and partly because it is unclear whether cell features of deeper structures render them magnetically silent (Hämäläinen et al., 1993).

Despite its well-characterized oscillatory properties (for reviews see Buzsáki, 2006; O’Keefe, 2007), it is often assumed that the hippocampus is difficult if not impossible to detect with MEG, an assumption which has only recently begun to receive critical reappraisal (Attal and Schwartz, 2013, Riggs et al., 2009). The hippocampus is a small curved bilateral structure constituting part of the archeo-cortex in the medial temporal lobe. It is ~5 cm long in adult humans (Schultz and Engelhardt, 2014), and in our simulations the distance between the centroid of the hippocampal mesh and the nearest sensor is 8.70 cm. Although it is thus deep relative to neocortical structures, it is more superficial than other structures successfully imaged empirically using MEG, such as the thalamus and brainstem (Attal and Schwartz, 2013, Coffey et al., 2016, Papadelis et al., 2012, Parkkonen et al., 2009, Wibral et al., 2013). Moreover, recent evidence suggests that the current source density generated by the hippocampal pyramidal cell layer is at least twice that of the neocortex, which might compensate to some degree for its distance to the sensors (Attal et al., 2012, Murakami and Okada, 2015, Murakami and Okada, 2006).

Indeed, cumulative evidence suggests that hippocampal sources can be identified in MEG, an observation made both in simulations (Attal and Schwartz, 2013, Chupin et al., 2002, Mills et al., 2012, Quraan et al., 2011, Stephen et al., 2005), and empirical data (Adjamian et al., 2004, Backus et al., 2016, Cornwell et al., 2012, Cornwell et al., 2008, Engels et al., 2016, Guitart-Masip et al., 2013, Hillebrand et al., 2016, Kaplan et al., 2012, Korczyn et al., 2013, Mills et al., 2012, Moses et al., 2011, Poch et al., 2011, Quraan et al., 2011, Riggs et al., 2009, Tesche and Karhu, 2000). Despite this body of theoretical support and empirical evidence, the sufficiency of the spatial precision of MEG for deep source reconstruction is still being debated (Mikuni et al., 1997, Mills et al., 2012, Riggs et al., 2009). One reason is that arguments for hippocampal involvement typically rely on the spatial location of a statistical peak in traditional group level volumetric inference. Consequently, factors which have led such findings to be toned down from ‘hippocampus’ to ‘medial temporal lobe’ include image smoothness at this depth (Gross et al., 2003), intra-subject variability, head movement, and in particular, co-registration error.

Another argument against hippocampal detectability is that its cylindrical geometry could cause signal cancellation (Baumgartner et al., 2000, Mikuni et al., 1997, Stephen et al., 2005). However, it has been demonstrated that the cancellation is lower than expected even when sources on opposing subfields are simulated (Stephen et al., 2005).

The aim of this paper is to demonstrate a method to infer not where an activation peak appears to be, but rather *whether* a model which includes the hippocampus does a significantly better job than a hippocampus-free model (i.e., a “null” model) at explaining activity from the hippocampus. We address this question by comparing two generative models: one comprised of the neocortex alone, and one which includes both the neocortex and hippocampus. A generative model is an account or hypothesis describing the putative origins of the measured signal. The models therefore enable formulation of competing hypotheses, and direct comparison hereof. This work echoes previous papers on the suitability of fMRI priors (Henson et al., 2009) and distinction between cortical laminae (Troebinger et al., 2014a), where for a given dataset we evaluate the evidence for two competing generative models. These models differ with respect to their anatomy, and therefore also with respect to their ability to account for the data when this portion of the anatomy is engaged. In this simulation study, we focus on explaining the method and testing its performance under different empirical constraints. We know from previous work that mesh-based generative models are extremely sensitive to co-registration error (Hillebrand and Barnes, 2011, Hillebrand and Barnes, 2003, López et al., 2012, Troebinger et al., 2014b) which therefore constituted our main factor of interest.

**Fig. 1:**
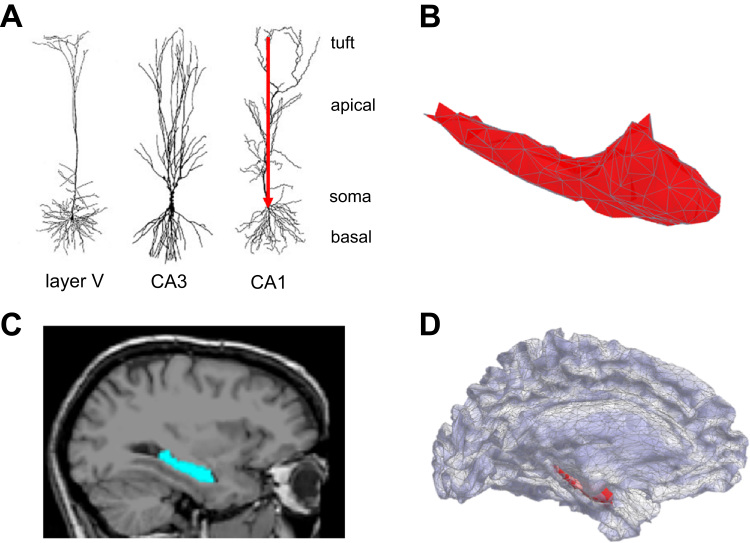
Hippocampal cell morphology, surface structure and location. **A** Morphology and similarity of pyramidal neurons in cortex and hippocampus. Postsynaptic potentials occurring at the apical dendrites or tuft give rise to the primary intracellular current (red arrow) which is measureable outside the head given a sufficiently large synchronously firing cell population. CA: Cornu Ammonis subfield of hippocampus. Cells pictured are from the rat. Image modified from (Spruston, 2008). **B** FreeSurfer-derived tessellated envelope of the left hippocampus. We model the sources to be perpendicular to mesh vertices, consistent with the pyramidal cell orientation. **C** Sagittal view of FreeSurfer hippocampal region of interest on a sample 1.5 T T1-weighted MR image from the FreeSurfer Image Analysis Suite. Blue colour shows the extent of hippocampal region of interest. Image adapted from (Hostage et al., 2013). **D** Source space representation of the combined generative model consisting of FreeSurfer-derived cortical (purple) and hippocampal (red) meshes. For more detailed description of this model, see Fig. 3. (For interpretation of the references to color in this figure legend, the reader is referred to the web version of this article.)

Here we propose an anatomically and electrophysiologically realistic generative model of hippocampal activity which accounts for geometry, depth and cell type. Through model comparison, this allows us to make categorical statements about which generative model is most likely for a given dataset – one with the hippocampus explicitly modelled, or one without. Although we focus on the hippocampus in this work, the approach should generalize to other brain structures with similar morphological and structural features. Here the modelling is motivated by the hippocampus' pyramidal cell layer's similarity to the pyramidal cell layer V in neocortex (which is the main generator of the MEG signal (Murakami and Okada, 2006)). Firstly, the pyramidal cells are morphologically identical in neocortex layer V and hippocampus (see Fig. 1A). Secondly, the pyramidal cell layer follows the surface curvature of the hippocampus, which means that it can be modelled as such. Thirdly, individual cells have dendritic trees oriented in parallel and with rich recurrent connectivity, causing magnetic fields to arise perpendicularly to the surface when synchronously active.

The main advantage of an explicit generative model is that it makes it possible to exploit not only the information from the estimated source location, but also its orientation (and other parameters such as current density and local coherence, although these are not considered here). We show that this allows us to differentiate the hippocampus from even the most proximal cortical sources.

In order to obtain probabilistic and comparative estimates of how good the two generative models are with respect to the data, we approximate their model evidence and compare the relative values in a Bayesian model comparison framework. This allows direct quantification of competing models’ abilities to explain the same data while avoiding over-fitting. Thus, building models equates to specifying prior beliefs about what could be expected from the data. In this case, the priors pertain to the anatomical locations and orientations of the potential sources. The priors can also pertain to functional properties of the sources, e.g. how the neural activity is structured, such as how sparse or smooth the sources are. These priors are specified in the form of different ‘functional’ priors or inversion schemes and we also test several of these to assess the robustness of our inferences across functional assumptions.

To approximate the model evidence for dataset inverted using a given model, we use Free energy (F), which is a lower bound on the true model evidence. Free energy rewards models which accurately fit the data, but penalizes models if they are overly complex (and therefore likely to over-fit). The logic in this context is that if electrical current was generated on the hippocampus but the hippocampus is not part of the generative model used to reconstruct the data, then a more extensive (and thus complex) mixture of cortical sources is required to explain the data equally well. Because of this increased complexity (see Wipf and Nagarajan 2009 on how the volume of the model covariance acts as penalty or sparsifying term), the cortical/hippocampus-free model will have a lower model evidence (or Free energy) value than the combined model which includes the hippocampus and therefore explains the data using fewer sources.

The paper proceeds as follows: we first describe the generative models, and then the simulation and source reconstruction parameters used. We then compare the two models across different scenarios with increasing co-registration error and signal-to-noise ratios (SNR). We do this across three different sets of popular functional priors: Minimum Norm Estimate (MNE), Empirical Bayes Beamformer (EBB), and Multiple Sparse Priors (MSP). This allows us to interrogate the model comparison framework from multiple angles, as there is no single superior functional prior since the performance depends both on the experimental question(s), performance criteria, and data (Hauk et al., 2011). Moreover, this allows us to address the consistency of results across functional assumptions.

## Methods

### Anatomical modelling of the hippocampus

The independent variable of our generative model is the hippocampal surface mesh. The pyramidal cells found in neocortex layer V and Cornu Ammonis (CA) subfields of the hippocampus are morphologically indistinguishable (Fig. 1A). In both pyramidal cell layers, the principal neuronal axes of the dendritic trees are arranged in parallel, and perpendicularly to the surface envelope. At a population level we therefore model current flow along the principal neuronal axis (red arrow) in the same way as per convention for the neocortex. Although the hippocampal pyramidal cells point in the opposite direction to those in neocortex, this does not influence the shape or extent of the magnetic fields produced and therefore need not be explicitly modelled. Thus, we constrain the sources to be oriented perpendicularly to the hippocampal mesh surface (Fig. 1B shows the surface envelope extracted from an MRI image). The hippocampus’ location is derived from the same anatomical MRI image as the neocortex. The hippocampus is shown overlaid on an MRI in Fig. 1C, and with respect to the extracted cortical mesh in Fig. 1D. As the hippocampus bulges into the floor of the (inferior horn of the) lateral ventricle, its medial surface extends more medially than that of the cortical surface. Apart from this, the hippocampus is nested inside the cortical manifold.

We extracted the left hemisphere's cortical and hippocampal surfaces for a single subject using FreeSurfer's (Reuter et al., 2012) automated image segmentation of individual T1-weighted MRI images (3 T Siemens Magnetom). We limited the simulations and re-constructions to the left side of the brain for simplicity. FreeSurfer gave a cortical mesh of the left hemisphere which we used directly, and a hippocampal volume file which we converted into a tessellated surface mesh. The resultant hippocampal surface was more densely tessellated than the cortical, so we smoothed and downsampled it such that the mean vertex-vertex distances were matched (mean values were 3.73 and 3.69 mm for the cortical and hippocampal meshes respectively). The number of vertices in the cortical and hippocampal meshes were 10595 and 162 respectively. This approach is consistent with the Deep Brain Activity model proposed by (Attal and Schwartz, 2013).

### Simulation set-up

**Fig. 2:**
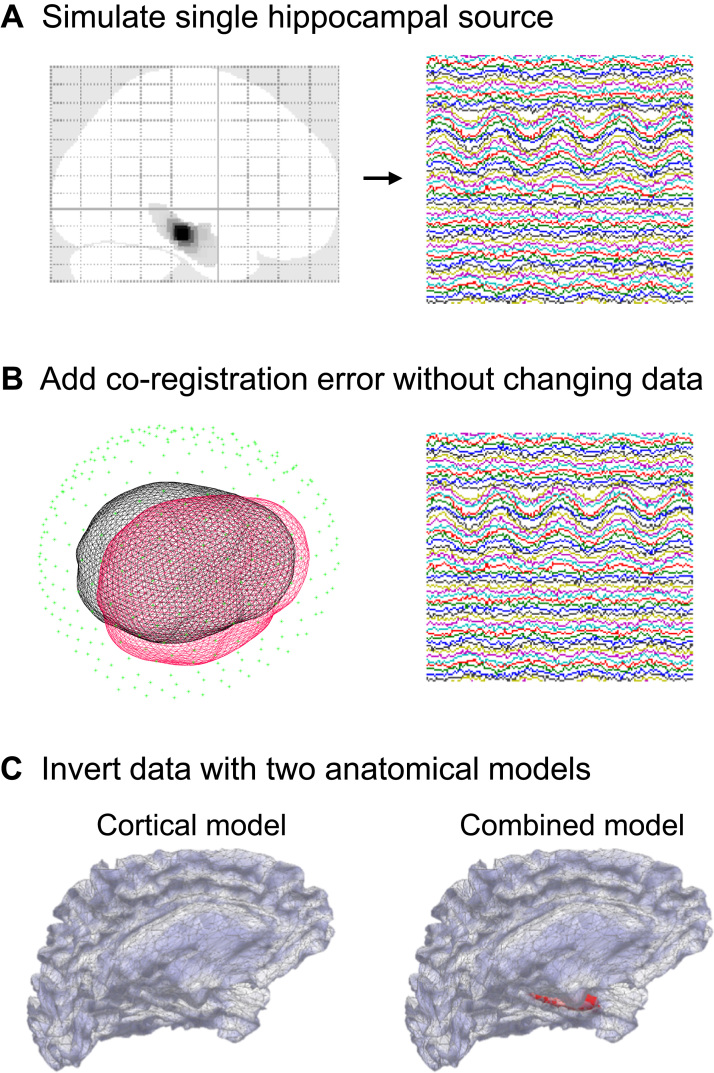
Overview of the simulation pipeline. **A** A single dipole source is simulated at a random location on the hippocampal surface as a temporal waveform with a sinusoidal frequency of 20 Hz. Gaussian white noise is added to the sensor level data (in this case −10 dB). On the right, a representative subset of the resulting 274 time-varying waveforms simulated are shown as coloured traces. **B** To simulate the effects of co-registration error, we add a displacement of 1, 2, or 3 mm standard deviation of error in each spatial dimension to each of the three standard fiducial points. The data themselves are left unchanged. The displacement shown here is exaggerated (2 cm) for illustration. **C** Next we invert the simulated data twice, using two different generative models. One with only the cortical surface (cortical model) and one with both cortical and hippocampal surfaces (combined model). We repeat this double inversion procedure on each dataset across three different reconstruction algorithms.

The simulation and reconstruction pipeline consisted of three steps: first, we simulated a single dipole perpendicularly to the hippocampal surface with a sinusoidal waveform of 20 Hz for 300 ms (six cycles) and a total effective dipole moment of 20 nAm (Fig. 2A). The simulation locations were randomly drawn from the 162 hippocampal vertices and were simulated with a full-width half-maximum of 6 mm. Each simulated dataset had a sampling rate of 600 Hz with the mean sensor-level SNR set to either 0, −5, −10, −15 or −20 dB, specified by adding Gaussian white noise to the data. We carried out 30 hippocampal and 30 cortical simulations at each SNR level (co-registration error is added at the inversion stage). This gave a core set of simulated data with known ground truth (i.e. whether or not the source was hippocampal).

In the second step (Fig. 2B), we mimicked the effect of co-registration error between functional (MEG) and anatomical (MRI) images by adding 0, 1, 2 or 3 mm standard deviations of error to each of three fiducial points in each of the three spatial dimensions. This shifted the surface mesh used for reconstruction (in red) relative to the surface mesh used to generate the simulation (in black). Co-registration error levels commonly seen empirically in MEG recordings are usually ~5 mm or more even with the best compensation tools, be they bite-bars (Adjamian et al., 2004, Singh et al., 1997) or algorithmic movement corrections (Whalen et al., 2008). However, using head-casts it is possible to bring it down to <1.5 mm (Meyer et al., 2017, Troebinger et al., 2014b).

After having perturbed the idealized data by adding sensor noise and co-registration error, we inverted the data using two different anatomical models and three different inversion schemes. One anatomical model was, per convention, just the cortical surface (Fig. 2C, cortical model), while the other model additionally included the hippocampal surface envelope (Fig. 2C, combined model). Each anatomical model was inverted using three different inversion schemes embodying functional (source covariance) assumptions. These were Minimum Norm Estimate (MNE) (Hämäläinen et al., 1993), Empirical Bayesian Beamforming (EBB) (Belardinelli et al., 2012) and Multiple Sparse Priors (MSP) (Friston et al., 2008a). We thus obtained six inversion solutions per simulated dataset; three inversion algorithms, each giving one solution per anatomical model. This lets us examine the difference between generative models across different assumptions about the nature of the activity – how sparse, how co-varying, how smooth etc. Each such inversion returns a Free energy value, which approximates the model evidence for generative model. This set-up allows us to quantify the difference in model evidence when the hippocampal mesh is included in the generative model. Our hypothesis was that there would be an improvement in model evidence only if the simulated source was hippocampal. This model comparison approach has successfully been demonstrated elsewhere (Henson et al., 2011, Henson et al., 2009, Lopez et al., 2013, López et al., 2014, Penny, 2012, Stevenson et al., 2014, Troebinger et al., 2014a). Here we use log Free Energy to quantify the difference between anatomical models: ΔF_anatomical_ = F_combined_ – F_cortical_. A positive difference means that the combined model is $\frac{1}{1+e^{∆F}}$ more likely than the cortical. If ΔF = 0 then the two models are equally likely, and if ΔF = 3 then the combined model is approximately twenty times more likely. All simulations and analyses were carried out using SPM12 http://www.fil.ion.ucl.ac.uk/spm/.

### Specification of anatomical priors

**Fig. 3:**
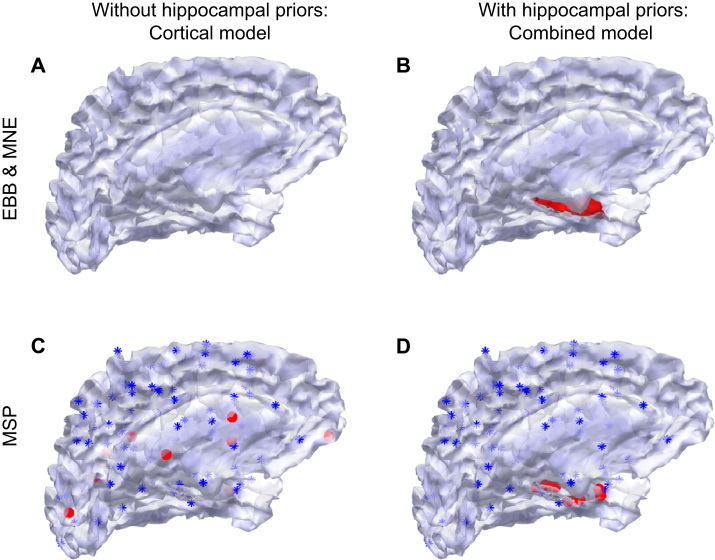
Anatomical models with and without hippocampal priors. **A** Implementation of the cortical model in the EBB and MNE algorithms. The tessellated cortical surface envelope is comprised of 10595 vertices. **B** Implementation of the combined model in the EBB and MNE algorithms. This model which includes a nested hippocampal manifold and contains 10757 vertices. **C** and **D** show the cortical and combined model implementations for MSP. The full source space is specified in both models such that each includes the nested hippocampal mesh and the number of vertices is 10757. Instead of vertices, the solution space is constrained by the spatial priors. In both models, 90 blue asterisks mark identical cortical prior locations. In **C,** an additional ten cortical priors are specified, marked here as red dots. In **D**, an additional ten hippocampal priors are specified, marked also as red dots. (For interpretation of the references to color in this figure legend, the reader is referred to the web version of this article.)

The schematic in Fig. 3 illustrates the two anatomical models and how they were implemented. The key difference is that MSP priors can be user-defined within subsections of the source space. Conversely, EBB and MNE make use of the complete source space. Left panels (A and C) show the cortical models and right panels (B and D) show combined models (with hippocampal priors). For EBB and MNE, the addition of hippocampal priors simply involves an addition to the source space which increases from 10595 vertices to 10757 vertices (Fig. 3A versus B). For MSP on the other hand, we kept the source space constant by using the combined model with 10757 vertices, but specified 100 spatial priors (patches of cortex) which either did or did not include the hippocampus. These spatial priors effectively constrained the solution space. The 90 blue asterisks mark cortical priors shared across the two models. The ten red dots distributed across the cortex mark cortical priors unique to the cortical model (Fig. 3C). The ten red dots on the hippocampus mark hippocampal priors unique to the combined model (Fig. 3D). Note that because we carried out 30 hippocampal simulations, we used three different sets of ten MSP hippocampal priors (keeping the 90 cortical priors constant). For each of these three sets of ten hippocampal priors, we matched the set of simulated hippocampal sources with the set of hippocampal priors . In all cases, we used a Nolte single shell (Nolte, 2003) to model the inner skull boundary.

The basic source code for the specification of anatomical priors, as well as simulation and reconstruction of the activity as outlined below is available on GitHub, as detailed in the Supplementary material alongside this article (see Appendix B).

## Source inversion

The empirical Bayes source inversion scheme has been described in detail elsewhere (Belardinelli et al., 2012, Friston et al., 2007, Henson et al., 2011, López et al., 2012, Phillips et al., 2005, Troebinger et al., 2014a). For a review, see (López et al., 2014). Here we elaborate on implementation issues and empirical applications (but see Appendix A for a more detailed account of how the algorithms used here differ with respect to specification of the prior source covariance).

All three algorithms require estimation of both a source and sensor level covariance matrix. In all cases we used an identity matrix to represent sensor level covariance as uncorrelated white noise. With respect to the source level covariance, the main difference between the three algorithms is that the MNE and EBB solutions require the optimization of a single source level covariance prior, whereas MSP requires optimization of multiple covariance priors. In MNE this matrix is also an identity matrix (such that one assumes that all sources have equal prior variance and are uncorrelated); whereas for the EBB algorithm it is derived directly from the data. For EBB and MNE, the algorithm must also estimate two (hyper) parameters which specify the weightings of the sensor- and source-level priors.. The MSP algorithm on the other hand takes a more general form and allows the source distribution to take the form of a combination of multiple source level covariance components. Each of these covariance components is a locally coherent patch of cortical activity.

The ensuing optimisation (to maximize Free energy) can be thought of as a process to minimize the number of patches while ensuring that the solution explains the maximum amount of data. This optimization consists of mixing and pruning of anatomical priors, which means that for large numbers of priors the optimisation can potentially get trapped in local extrema. One practical solution to avoid this is to run the same algorithm many times with different sets of priors (Troebinger et al., 2014a). However, as we were not interested in the optimisation per-se, but rather in finding the best possible solution, we used (only) 100 priors for MSP and simulated sources at a subset of these locations. Note that there was thus a clear advantage for the MSP algorithm relative to EBB and MNE, because the best solution is fixed to lie in the space of MSP priors, which is much smaller than the EBB or MNE space (i.e. all the vertices; see Fig. 3). This advantage is elaborated upon in the discussion and is relevant for both hippocampal and cortical simulation results. More specifically, in case of the hippocampus, the ten MSP priors included in the MSP generative model always include the patch used the generate the simulated dataset. Although the simulation patch is also included in the EBB/MNE combined generative model, the latter also includes the remaining 161 hippocampal vertices in the hippocampal mesh. Similarly, for the cortical simulations, the solution space was defined by 90 anatomical priors for MSP, again including the simulated patch(es), versus all 10595 cortical vertices specified for the EBB/MNE algorithms. Importantly, in addition to the anatomical model comparisons, it is also possible to directly compare the inversion schemes by keeping both the data and model constant, and varying the algorithm (see Supplementary Figure 4).

**Fig. 4:**
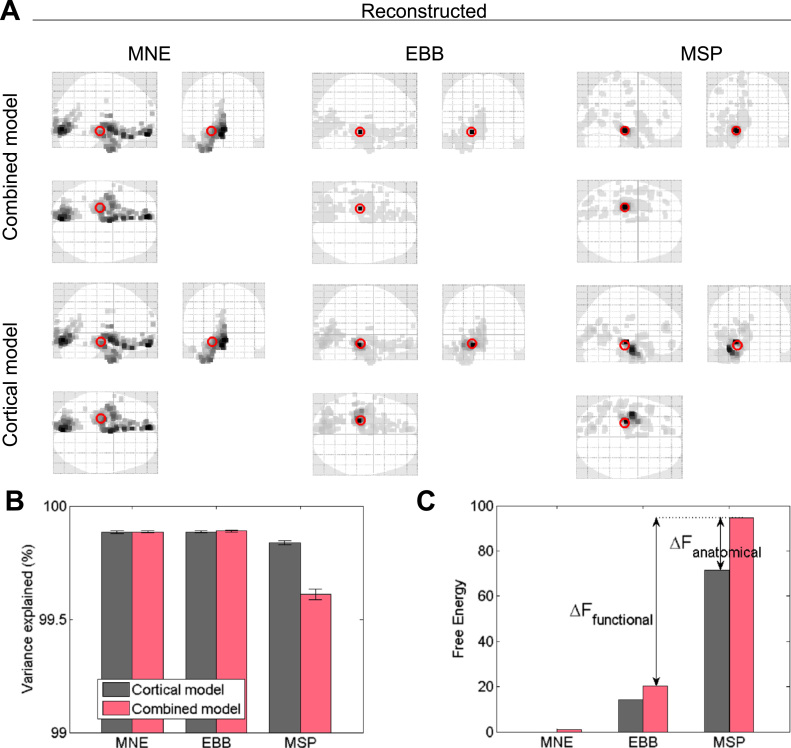
Sample source reconstructions and model comparison. **A** Single-trial reconstructions of a hippocampal source (red circles) with MNE, EBB and MSP priors using the combined model (top row) and the cortical model (bottom row). EBB and MSP accurately capture the true source location. Glass brains show estimated current source density with the grey scale proportional to the darkest (maximally active) vertex location. Sample source simulated with SNR −5 dB and no co-registration error. **B** Variance explained by different anatomical and functional priors when simulated sources are hippocampal. Bars encode mean percentage variance explained across 30 hippocampal simulations (±SEM). Note that the y axis only spans 99–100%. For this metric there were no significant differences between models with EBB (t(29) = 1.0842, p = 0.287) or MNE (t(29) = 0.1591, p = 0.875). For MSP in contrast, there was a significant difference in the percentage variance explained (t(29) = −8.6310, p < 0.001), but favouring the incorrect (cortical) model. **C** Same as B, but showing Free energy values and Bayesian model comparison methods. Free energy (F) is used to approximate the model evidence of a given solution. Bars encode mean Free energy values over 30 simulations, normalized to MNE cortical. Differences between anatomical priors we denote ΔF_anatomical_ whereas differences arising from different functional priors we denote ΔF_functional_. (For interpretation of the references to color in this figure legend, the reader is referred to the web version of this article.)

We did not use any spatial dimension reduction (i.e. all 274 functioning MEG channels were used) but we decomposed the time series into a single temporal mode. We used three different forms of functional priors (MNE, EBB and MSP) and two sets of anatomical priors (cortical versus combined model). Sample inverse solutions for all six prior combinations are shown in Fig. 4A. We carried out 30 iterations of each hippocampal and cortical simulations at each SNR level.

### Dipole localisation error

In order to provide a frame of reference between the model evidence-based approach used here, and those approaches other simulation studies we also calculated the dipole localisation error (DLE). The DLE equates to the distance between the true simulation location and source distribution maximum of the inversion. We calculated DLEs separately for the combined and the cortical models used to invert 30 hippocampal and cortical simulation scenarios using EBB.

## Results

### Variance explained and free energy

In order to demonstrate the basic logic behind our analysis Fig. 4A shows a representative single-simulation source reconstruction for each combination of anatomical and functional priors. We can compare the algorithms qualitatively with respect to accuracy and complexity because we know the true source location. First, spatial accuracy can be assessed by looking at how far the simulation vertex (red circle) is from the peak (darkest vertex) of the estimated current distribution. Second, complexity is reflected in the spread of the source estimates. Note that when the correct anatomical model is used (Fig. 4A, top row), for EBB and MSP, the source estimates are generally accurate and focal. The increase in spatial spread or complexity (most noticeable for MSP and EBB) in the bottom row (inversions using just the cortical model) occurs because it requires more non-hippocampal sources to describe MEG data arising from a single hippocampal source than would be needed if the true source were modelled.

We find that as expected, MNE gives the most diffuse solution and MSP and EBB give the most focal. Nonetheless, it is encouraging to note that although the algorithms have different functional assumptions, the estimated activity is in approximately the same place throughout.

In contrast to Free energy, percentage variance explained is not penalized for complexity and consequently is not discriminative of the correct model. Fig. 4B illustrates the mean percentage of variance explained for the two models across 30 iterations of hippocampal simulations with SNR −5 dB while Fig. 4C illustrates the mean Free energy. Note that the mean variance explained is >99.5% for all algorithms, and that the best model in terms of Free energy (MSP) does not explain the most variance. This is because there is less over-fitting of the noise.

Given that the Free Energy value does not rely on information about the true source location, it is ideally suited not only for simulated data to avoid over-fitting, but particularly for empirical data where the true source location is now known. Furthermore, it has been shown previously that Free energy correlates with cross-validation accuracy (Penny and Roberts, 1999), and with conventional reconstruction evaluation measures such as dipole localization error (Belardinelli et al., 2012). Thus, although we *do* have access to the ground truth in these simulations, we will nonetheless rely on Free energy as a goodness of fit criterion while also evaluating the dipole localisation error for comparison. The main focus will be evaluation of anatomical and functional Free energy (F) differences, calculations of which are shown in Fig. 4C. We first compare anatomical priors by subtracting the two F values obtained using different anatomical models with the same algorithm. This is shown for MSP where ΔF_anatomical_ = F_combined_ – F_cortical_. We then compare functional priors by subtracting the two Free energy values obtained using the same anatomical model (e.g. the combined model) but different algorithms, e.g. ΔF_functional_ MSP vs EBB = F_MSP_ – F_EBB_. This metric tells us how good the functional assumptions are (how smooth/sparse etc.), because the data and anatomical model are constant (the results of these tests are shown in Supplementary Figure 4).

The main emphasis of this paper is on ΔF_anatomical,_ and thereby quantifying hippocampal engagement probabilistically through comparison of generative models. With respect to single-simulation ΔF_anatomical_ values corresponding to solutions shown in Fig. 4A_,_ we find that for all three algorithms, the combined (true) model has a higher Free energy value than the cortical model; single simulation ΔF_anatomical_ MNE = 1.4, EBB = 10.6, MSP = 73.2. We find that the average ΔF_anatomical_ values across 30 simulations (Fig. 4C), are lower but somewhat similar (mean ΔF_anatomical_ MNE = 1.0, EBB = 6.0, MSP = 23.1). Note that only EBB and MSP pass the significance threshold of three (log units) where the combined model is 20 times more likely than the cortical. Thus, even without knowledge about true simulated source locations, Bayesian model comparison can be used to distinguish whether the source location is hippocampal or not based on the model evidence difference. Interestingly, in this example EBB appears (from the source level maps) to perform equally well for both anatomical models. One explanation for why the peak of the cortical model solution appears to be in/on the hippocampus when it is not explicitly modelled (Fig. 4A), is that the cortical and hippocampal mesh surfaces are very close together (few mm on average, see Fig. 3B). Since EBB can distribute variance across all source vertices, those on the medial temporal lobe could therefore appear hippocampal. This issue is directly addressed later in Fig. 8.

### Anatomical model comparison

**Fig. 5:**
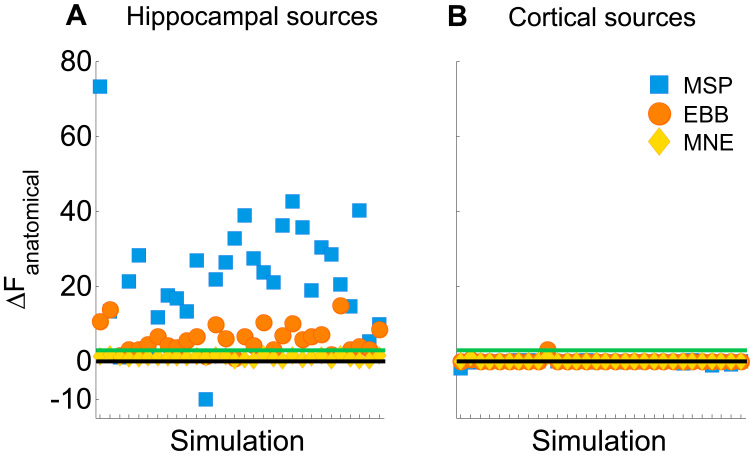
Anatomical model comparison for hippocampal and cortical (control) sources. **(A)** Dots show ΔF_anatomical_ = F_combined_ - F_cortical_ values for sources simulated on the hippocampus. ΔF_anatomical_ is positive because the combined model explains more data using a simpler solution (fewer hippocampal priors). The black line marks zero where there is no difference between models. The green line marks a positive difference of three which, because Free energy is on a log scale, means that the combined model is >20 times more likely than the cortical. MSP outperforms the other algorithms while MNE fails to reach significance. **(B)** Shows the results for the simulated cortical sources or control condition (note that exactly the same comparison between full and cortical models is made). There is little if any difference between models because the two generative models contain the same cortical mesh (all 10595 cortical vertices for EBB and MNE) or cortical priors (90/100 priors for MSP where the hippocampal priors are redundant and therefore pruned away in the combined model). For the 30 hippocampal and 30 cortical simulations shown, SNR is −5 dB and no co-registration error is added.

We evaluated two variations of the same basic generative model, one that includes a nested hippocampal manifold and one which does not. To first verify that the combined model helps to explain hippocampal activity, we simulated 30 hippocampal sources and compared the Free energy values obtained with the two anatomical models for each simulated dataset (ΔF_anatomical_ = F_combined_ – F_cortical_). Fig. 5A shows the positive ΔF_anatomical_ values from across 30 simulated hippocampal datasets with SNR of −5 dB and zero co-registration error. As a first control, we then tested whether this Free energy difference was specific to hippocampal activity, or could be driven simply by an increase in vertices in the combined model, regardless of the location of the source. To test this, we simulated sources on the cortical surface and evaluated them in the same way as before. The prediction is that if the hippocampal mesh is selectively beneficial only when evaluating hippocampal sources, and not generally introducing bias, then there should be no difference between models in the case of cortical sources. The cortical sources simulated to test this were randomly distributed across the cortical mesh and again the simulation locations equated to (30 of the) cortical MSP priors. Given that the locations of the cortical priors (sparse in the case of MSP and mesh-wide in the case of EBB and MNE) were identical in the cortical and combined models, we expected to find no difference in F between anatomical models. Fig. 5B shows the null ΔF_anatomical_ values for data simulated on the cortical surfaces.

### Effect of co-registration error

**Fig. 6:**
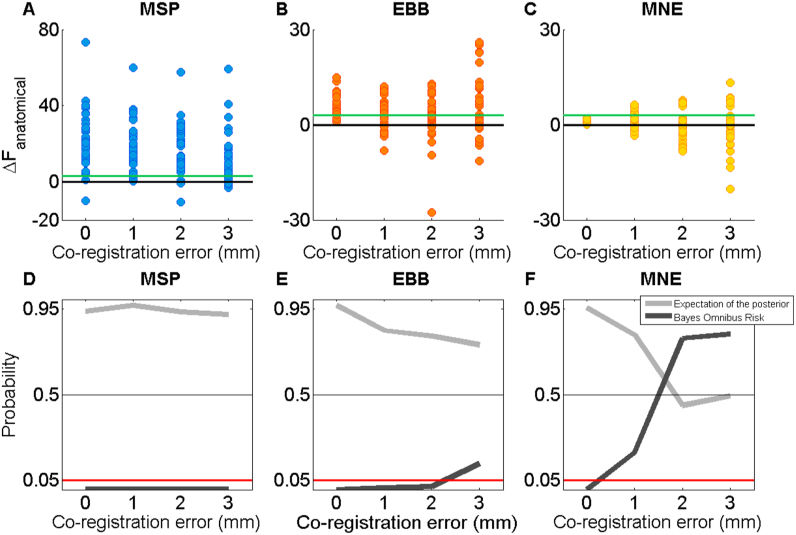
Effect of co-registration error on anatomical model comparison. Inversion results from simulated hippocampal dipoles with SNR −5 dB and co-registration error simulated as 0, 1, 2 or 3 mm standard deviation of error added to each of the three fiducial locations in each dimension. **Top panel (A-C)**: Dots represent ΔF_anatomical_ for the same 30 simulations at each co-registration error level. There is an increased spread of values, and an increased number of negative ΔF_anatomical_ values (false negatives) as a function of co-registration error. Green line marks the significance threshold of three, black line marks no difference. Y-axes of EBB and MNE plots are adjusted for visibility. **Lower panel (D-F)** is structured in the same way as upper panel but depicts two measures of the reliability of the model comparisons shown above. Light grey line marks the expectation of the posterior; the probability that the combined model supersedes the cortical model. Dark grey line marks the Bayes Omnibus Risk (BOR), the probability that there is no difference between models. We can reject this null when the BOR metric is below 0.05 (red-line). (For interpretation of the references to color in this figure legend, the reader is referred to the web version of this article.)

We then examined the effect co-registration error on our ability to identify the correct (combined) model when sources were hippocampal. To do this, we simulated co-registration error by adding 1, 2 or 3 mm standard deviation of error to each of three fiducial locations in each of three dimensions before inverting the model (see Fig. 2B). Note that the shift and data were always the same for the two models which therefore only differed with respect to the inclusion of a hippocampal mesh. Fig. 6A-C shows the Free energy differences obtained for the 30 hippocampal simulations described previously but with different levels of co-registration error, including zero. As expected, the difference values decrease as co-registration error increases, demonstrating that uncertainty about head location compromises our ability to discriminate between models. We also found that the variability of the Free energy differences increases, illustrated most clearly with MNE (Fig. 6C).

To quantify the decrease in reliability of the results as a function of increased variability, we used a random effects analysis (Stephan et al., 2009) to estimate the probability that the correct (combined) model would win given a randomly drawn simulation (light grey lines, Fig. 6D-F). Consistent with the Free energy difference decreases in the top panel, this probability decreases as co-registration error increases. Thus, if we were to select a dataset at random, we would expect to make the correct decision (i.e. identify the combined model as better and infer the presence of a hippocampal source) ~95% of the time with MSP, regardless of co-registration error. With the EBB this chance would decrease to ~75% at 3 mm of error and with MNE, we would be at chance level with 2 mm of error. One interesting but subtle problem with this inference is that there is an underlying assumption that one model is better than another; that the model frequencies across this set of simulations are not equal. In order to derive a conservative bound on where the models truly differed, we therefore computed the Bayes Omnibus Risk (BOR) which quantifies the probability that the null hypothesis is true and any observed differences between models are due to chance (Rigoux et al., 2014). BOR probabilities (dark grey lines in Fig. 6D-F) of less than 0.05 (red lines) mean that the null hypothesis can be rejected. This analysis shows that even 3 mm of co-registration error (which is far less than what is commonly found in standard experimental set-ups) abolishes our ability to distinguish between anatomical models with EBB and MNE. With MSP, there is still a reliable difference between models at 3 mm of co-registration error, but the priors are well-known (which is not realistic empirically). In sum, increased co-registration errors of ~3 mm or larger blur out existing differences between the anatomical models. Note that the closer the functional prior to the ground truth (compare MSP and MNE), the more robust it will be to co-registration error.

### Effects of co-registration error and sensor-level SNR

**Fig. 7:**
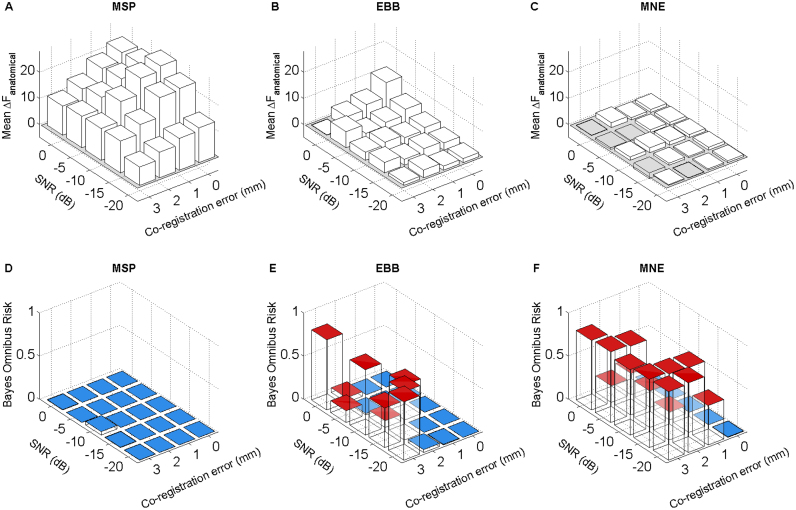
Effects of noise and co-registration error on anatomical model comparison. The figure is similar to Fig. 6 with an added dimension of noise. **Top panel (A-C)** shows negative effects of co-registration error and noise: ΔF_anatomical_ decreases as a function of both (and of either alone). Each bar encodes average ΔF_anatomical_ of 30 reconstructed hippocampal simulations. **Lower panel (D-F)** shows roughly the same effects on the Bayesian Omnibus Risk, the probability that anatomical model frequencies are equal. Co-registration error above 0 and 1 mm are detrimental for MNE and EBB model comparisons respectively. Bar top colours signify when the null hypothesis, that there is no difference between models, can be rejected (BOR values <0.05, blue bar tops) and not rejected (BOR>0.05, red bar tops). (For interpretation of the references to color in this figure legend, the reader is referred to the web version of this article.)

We next investigated the interaction between sensor level noise and co-registration error. We added different amounts of uncorrelated white noise to obtain 0, −5, −10, −15 and −20 dB SNR at sensor level. Fig. 7 takes the same form as Fig. 6 but includes an added SNR dimension. The upper panel (Fig. 7A-C) shows mean ΔF_anatomical_ over 30 hippocampal simulations where positive values show evidence in favor of the combined model. As expected, we find that as both co-registration error and noise increase, ΔF_anatomical_ decreases. The lower panel (Fig. 7D-E) shows the Bayes Omnibus Risk (BOR) quantified based on 30 hippocampal simulations at each combination of SNR and co-registration error. Blue bar tops mark values BOR<0.05 where we can reject the null hypothesis that the models are equivalent, red bar tops indicate no significant difference between models (even if the Free energy difference is significant on average). In general, we find that poor SNR is less detrimental to our ability to differentiate models than co-registration error is (seen most clearly with MSP). As before, we conclude that co-registration error must be <3 mm to make reliable identification of hippocampal activity with EBB and MNE. As expected (or defined by our simulations), the MSP outperforms the other two algorithms at all levels of co-registration error and SNR tested here. See Supplementary Figure 2 for the same analysis using the Low Resolution Electromagnetic Tomography (LORETA) method.

### Closest cortical neighbours

As spatial resolution decreases rapidly with depth in MEG, there is a risk that higher Free energy values for the combined model could arise from nearby but non-hippocampal sources, yet be misinterpreted as hippocampal activity through this inference scheme. In other words, one might worry that medial temporal lobe sources will cause false positive results.

**Fig. 8:**
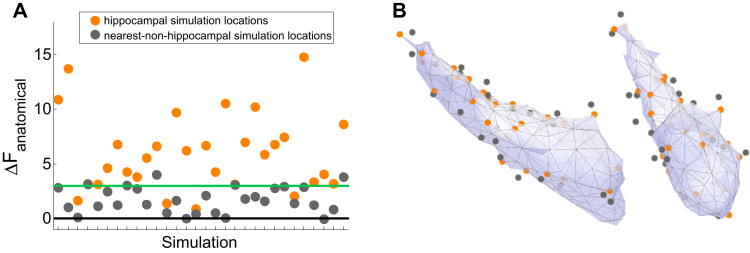
Closest cortical neighbour control analysis: activity is simulated on closest neighbouring cortical vertex for each hippocampal vertex location used as a source. **(A**) Dots reflect the Free energy difference (ΔF) when activity is simulated on the hippocampal mesh (orange dots; 30 different sources) and nearest cortical vertex (grey dots; 30 different sources). Dots are vertically aligned in pairs of closest neighbours. Simulating hippocampal simulations sources gives significant (>3, green line) ΔF values, whereas simulating on the nearest cortical neighbour generally does not. Parameters used were no co-registration error, SNR −5 dB and EBB. **(B)** Simulation locations visualised on two views the hippocampal mesh. Orange dots are on the hippocampal surface, grey are on the cortical surface (not visualised). Average distance between closest neighbours is 2.14 mm. (For interpretation of the references to color in this figure legend, the reader is referred to the web version of this article.)

We tested this by simulating activity on the nearest cortical vertices to each of the 30 hippocampal vertices used in the original simulations and inverting these data with both the cortical and combined models to calculate the Free energy difference for each cortical location. Reassuringly, we found the average Free energy difference for the closest cortical neighbour simulations to be 1.75 and thereby non-significant (Fig. 8A**,** grey dots). Conversely, the hippocampal simulations gave positive and significant (ΔF>3, mean = 6.0) evidence in favour of the combined model (as shown in Fig. 5). Critically, the average distance between neighbouring hippocampal and cortical vertices was only 2.14 mm (Fig. 8B). We focused here on EBB because its performance was mid-range and because it does not require specification of priors.

### Effects of shifting the hippocampus

**Fig. 9:**
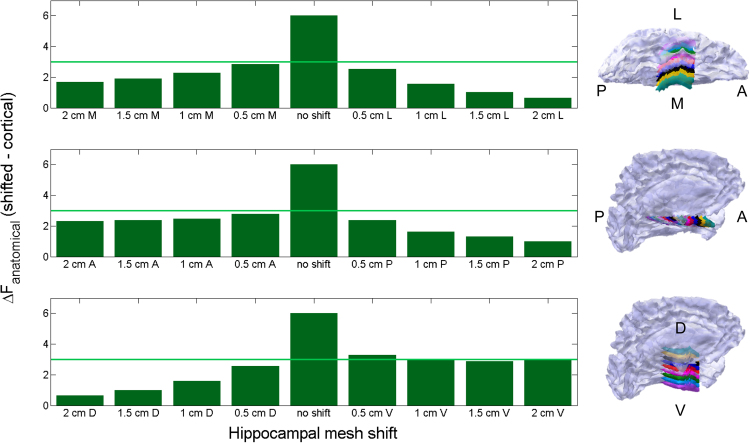
Effect of shifting the hippocampal mesh on Free energy. We compare different combined models with shifted hippocampal meshes to the standard cortical (hippocampus-free) model. Bars represent average Free energy differences (mean F_shifted_ – F_cortical_) across 30 different hippocampal simulations. Top panel shows medial-lateral shifts, middle panel anterior-posterior, bottom panel dorsal-ventral. While the no shift comparison (standard combined – cortical) gives a significant average Free energy difference (>3), shifting the hippocampus in any dimension or direction renders the model comparison non-significant (difference <3, except for 0.5 cm lateral). Light green line at F=3 marks the significance threshold where the combined (with or without hippocampal shift) model is >20 times more likely than the cortical.

To ensure that the Free energy differences were specific to the correct model and not simply to having a deep structure added, we carried out a set of inversions with models that had the hippocampus (slightly) offset relative to the correct location. For this analysis, we used the same simulated hippocampal data as described previously (i.e., activity simulated on the hippocampal surface in its original location), but inverted these data using combined anatomical models with the hippocampal mesh slightly offset from the correct location (0.5, 1, 1.5 and 2 cm shifts) in three dimensions (medial-lateral, anterior-posterior, dorsal-ventral), and two directions (positive and negative) giving 24 different models with a shifted hippocampus (Fig. 9). In all models, the cortical portion of the combined model stayed the same. As with other control analyses, we focused here on EBB and used simulations with SNR −5 dB and no co-registration error. We inverted each of the 30 datasets with each of the 24 shifted models and compared the resulting Free energy values to those obtained with the standard cortical model as well as standard combined model. Only in cases where there is no shift (i.e. the correct combined model is used, middle bars), is the model comparison with the cortical significant. This demonstrates specificity of the model comparison approach to correct hippocampal modelling, and the ability of this approach to identify the correct model among a set of subtly offset alternative models. In other words, despite the physical overlap between the cortical and hippocampal surfaces when the hippocampus is shifted, the disparity in the surface orientations mean that these shifted surfaces are poor generative models compared to the correct one. Supplementary Figure 1 shows the opposite comparison to Fig. 9; ΔF = F_standard combined_ – F_shifted_. This comparison shows that the standard combined model is significantly better than the shifted. See also Supplementary Figure 3 for a similar approach where the hippocampus is rotated instead of shifted but results are similar.

### Dipole localisation error

**Fig. 10:**
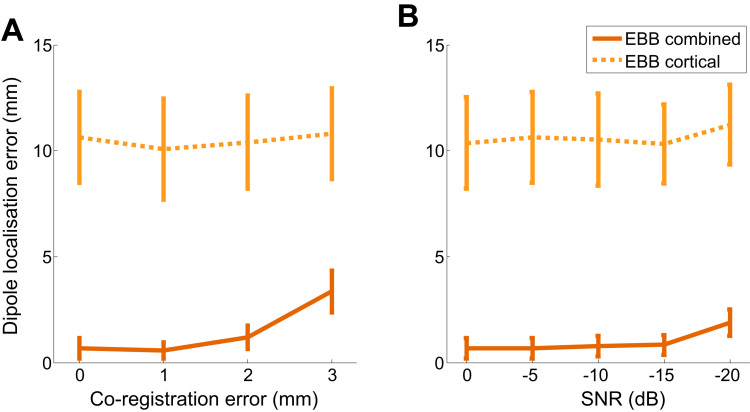
Dipole localisation errors as a function of co-registration error and SNR when sources are hippocampal. **A** Mean dipole localisation error (±SEM) against co-registration error. SNR −5 dB. Dotted yellow lines show results for EBB using the cortical model; orange solid lines used for combined. The cortical model gives higher and more varied DLE values than the combined. For the combined model, DLE and variability starts to increase when co-registration error exceeds 1 mm. **B** Mean dipole localisation error (±SEM) across SNR levels. Again the cortical model gives higher and more varied DLE values irrespective and does not vary with SNR. For the combined model, both error and variability increases when sensor-level white noise exceeds −15 dB. No co-registration error added. (For interpretation of the references to color in this figure legend, the reader is referred to the web version of this article.)

We also performed a more traditional analysis by calculating the dipole localisation error (DLE) between the simulated and reconstructed sources (Fig. 10). As expected, both the average DLE and its variance increases as co-registration error increases (Fig. 10A) or SNR decreases (Fig. 10B). Furthermore, we found that in accordance with our Free energy results (Fig. 6, Fig. 7), DLE is more affected by co-registration error than by SNR. By definition, DLE can only be calculated when the true source location is known, i.e. in simulations. Critically therefore, the correspondence between the DLE and Free energy supports the notion that Free energy is valid and informative when the true source location is not known, i.e. in empirically recorded data.

Furthermore, we quantified how often the hippocampal simulations have source distribution maxima on the hippocampal mesh (the true positive rate or sensitivity), and how often cortical simulations have maxima on the cortical mesh (the true negative rate or specificity). At SNR −5 dB and no co-registration error, we find that the sensitivity is 93.33% and specificity is 100%.

### Multiple sources

**Fig. 11:**
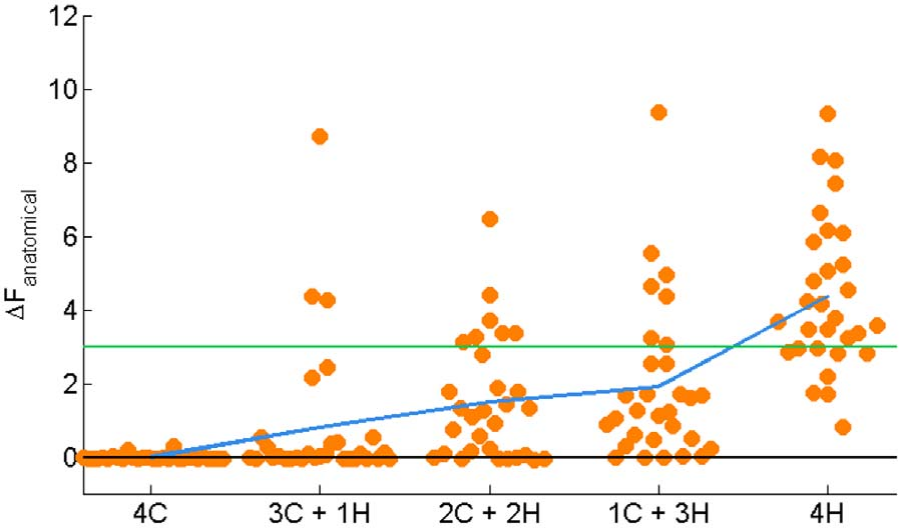
Multiple sources simulation. To test whether the model comparison framework would generalise with more than one dipole, we simulated four simultaneous dipoles at different ratios of sources on cortex (C) and on hippocampus (H). Orange dots at each ratio of source locations represent 30 Free energy differences for EBB inversions. The blue line marks the mean Free energy difference at each proportion of hippocampal dipoles. As the proportion of hippocampal sources increases, the mean Free energy difference increases. This mean difference only reaches significance (>3, green line) when all four dipole locations are hippocampal. Each source was simulated with band-limited white noise waveforms between 1–80 Hz for 300 ms and the effective dipole moment was set to 100 nAm for cortical sources, and 200 nAm for hippocampal sources The simulation locations were the same as used previously (which were drawn at random). Each simulated dataset had a sampling rate of 600 Hz with the sensor-level white Gaussian noise level now defined as an absolute value of 10 root mean squared (rms). Due to the range of frequencies simulated, we used 16 temporal modes (as opposed to a single mode previously) to describe the data. We added no co-registration error to these inversions. (For interpretation of the references to color in this figure legend, the reader is referred to the web version of this article.)

One further question is whether this approach is robust to situations containing a mixture of cortical and hippocampal sources. Fig. 11 shows the relationship between ratio of cortical-to-hippocampal sources, and Free energy differences between the combined and cortical models. As expected, the greater the proportion of sources within the hippocampus, the greater the model evidence in favour of the combined model. However, the mean Free energy difference only reaches significance (>3) when all four dipoles are hippocampal. Note also that we simulated hippocampal sources with twice the dipole moment as cortical sources to reflect the higher density of the pyramidal cells in this structure (Attal et al., 2012, Murakami and Okada, 2015, Murakami and Okada, 2006). Importantly this analysis also reassures us that (multiple) purely cortical sources (condition 4 C) do not lead us to infer, incorrectly, that the hippocampus was involved (Fig. 5B).

## Discussion

We show that it is possible to reliably infer specifically hippocampal (rather than medial temporal lobe) activity through comparison of two generative models, one with and one without the hippocampus explicitly modelled. For this inference to be reliable, uncertainty about the location of the brain relative to the sensors must be minimized to below 3 mm.

### Bayesian model comparison

The approach presented here works on the basis of the following rationale: a generative model of the data with the hippocampus explicitly modelled will be better at explaining hippocampal activity in the sense that it provides a more parsimonious solution than would a model without the hippocampus. Consequently, this model will be penalized in terms of its model evidence. Therefore, although the cortical and combined models may explain the same amount of variance in the data (Fig. 4B), the cortical model must use more sources to do so, resulting in a lower Free energy value (Fig. 4C).

The most immediate advantage of the Bayesian model comparison method is that it allows us to make use of much more prior information when making the same inference. For example, instead of simply looking at the location of the peak in an image, we can use a generative model to test whether the orientation of the source is what we would have expected.

This raises the question of what the level of detail required in the hippocampal model is for evaluation of empirical data. It would be interesting for example to test whether for such data we can distinguish between canonical and individual models of the hippocampus (similar to work on the cortex, see (Henson et al., 2009; Troebinger et al., 2014a)). We are so far encouraged by the sensitivity of our inference to hippocampal location (Fig. 9**,** Supplementary Figure 1) and orientation (Supplementary Figure 3). Here we have focused on the distinction between cortical and hippocampal surface sources (Fig. 5**,** Fig. 8) but we hope to eventually incorporate structural features of hippocampal subfields and close-by structures (retrosplenial cortex, parahippocampal cortex, entorhinal cortex, amygdala, etc) into the modelling of neuronal current flow. This would allow the uncertainty to be further reduced and for us to begin to distinguish between hippocampal subfields and different subcortical structures in MEG, and thereby begin to study their real-time interactions non-invasively and with a temporally resolved method.

The approach we have presented relies heavily on Occam's razor - more parsimonious models will always be favoured if they explain the same data. For example, see Fig. 4 where the combined model represents a simpler solution and is therefore favoured (Fig. 4**C**, F_combined_ > F_cortical_), even though it explains less variance (Fig. 4**B**). Similarly, we are consistently able to distinguish the true hippocampal source from the array of sources in the temporal lobe (Fig. 8) due to the increased simplicity of the solution obtained using the correct (combined) model. An important caveat is therefore that were the true source pattern to be distributed over medial temporal lobe and produce measured data consistent with a single hippocampal source, our method would erroneously categorize this activity as hippocampal.

Although the spatial resolution is inevitably poorer at deep locations in the brain (Hillebrand and Barnes, 2002), we have shown that the approach presented here is sensitive enough to discriminate between hippocampal and neighbouring cortical sources, even when these are as close together as ~2 mm (Fig. 8). We attribute this discriminability to the different orientations of the local surfaces which give us leverage to distinguish between models not commonly available in more traditional voxel-wise inference where only location information can be used. As such, Bayesian model comparison is distinct, and complementary to standard group level voxel-wise statistics in which we traditionally look for a peak location within a specific structure. The key difference here is that for each subject we have anatomical models which constrain not only source locations but also orientations (and potentially in the future also expected current densities (Helbling et al., 2015)) which give us an extra dimension with which to distinguish between models.

### Assumptions, implications, and limitations of simulation choices

We simulated data using one set of functional priors (suited to MSP) and reconstructed data using this and two other commonly used covariance assumption sets (beamforming and minimum norm). The MSP performs the most robustly and sensitively of these three. This is unsurprising, given that the simulated activity was sparse, a characteristic that matches with the MSP assumptions. Another important point is that we pre-selected the correct set of priors (spatial patches) for MSP and therefore side-stepped a potentially computationally intensive search over possible patches which would be necessary for empirical data (for example see (Troebinger et al., 2014a) where we used 32 random patch sets per dataset and cortical model). This means that while MNE and EBB had the same large search space, only MSP was given priors to start the search from which exactly matched the actual simulation location.

Overall we were encouraged to find that all three algorithms showed a preference for the correct anatomical model (Fig. 3B-D, Fig. 9**,** Supplementary Figures 1 and 3) and gave somewhat similar estimates of the true source distribution (Fig. 4A). Importantly, as the true functional priors will never be known, the Free energy equation also allows us to select the most likely functional priors (Supplementary Figure 4). Given that the EBB algorithm did not have the advantages of the reduced MSP prior space, yet performed well, and given the wealth of previous hippocampal studies using (volumetric) beamformers (Cornwell et al., 2012, Guitart-Masip et al., 2013, Kaplan et al., 2012, Poch et al., 2011), we think this is a promising avenue for further work.

We note that the inversion algorithms used here are somewhat generic and not individually optimized. For example, many centres define a baseline period or empty room recording which allows an estimate of the optimal regularization parameter. Likewise, there is no depth re-weighting in the MNE estimates. Here all regularization (the balance between the source and sensor level covariance matrices) was set based on a Free energy optimization (Friston et al., 2008a). We also make no use of any dimension reduction on the lead-field structure or the data (Engemann and Gramfort, 2015, Friston et al., 2008a). We avoided this to remove any bias between competing models with different lead-field structures. We have also used rather fine meshes (10757 vertices for the combined model which includes one hemisphere and one hippocampus) as compared to those commonly employed in packages such as Freesurfer (https://surfer.nmr.mgh.harvard.edu/) or MNE (Gramfort et al., 2014): MNE uses Freesurfer meshes and gives users the choice of mesh density: 1026, 2562, 4098 (used as example), or 10242 sources per hemisphere.

It is also important to consider the main limitations and assumptions related to using Bayesian model comparison and Free energy. Firstly, as is true for any model comparison scheme, we cannot evaluate how good the individual models are in absolute terms; we can only infer how good they are relative to one another. It is therefore not possible to make inferences or predictions about whether alternative models might be better without constructing and testing such models. It follows that if the true activity arises from a neighbouring structure (such as the amygdala), but we have not included a model of the amygdala in the generative model, then we may make an incorrect inference.

However, we found evidence that the models employed here perform well both in terms of Free energy, and in terms of identifying the correct simulated source location (Fig. 4 and Fig. 10). More specifically, while both models perform well, the combined model performs better. Nonetheless, there is a risk of having local maxima in the cost function (in this case the Free energy) if the number of sources and/or hyper-parameters is very large (Wipf and Nagarajan, 2009). This would mean that models could converge on non-optimal solutions and thereby render the F value an invalid reflection of the model or algorithm's optimal parameter settings. That said, it has been shown elsewhere using simulated data that Free energy correlates with cross-validation accuracy using machine learning approaches (Penny and Roberts, 1999), and with conventional reconstruction evaluation measures such as dipole localization error (Belardinelli et al., 2012). We also find this to be true in our data (Fig. 10). It follows that maximization of Free energy can be used to fine-tune features of the generative model used for analysis, such as number of equivalent current dipoles (Kiebel et al., 2008), forward model (Henson et al., 2009), or cortical layer giving rise to the measured signal (Troebinger et al., 2014a). However, perhaps the greatest advantage of Free energy is that it provides a framework for reliably evaluating hypotheses without knowledge of ground truth.

As with all simulations, these data represent well-defined perturbations to an ideal situation – as such, the estimates obtained (such as how much co-registration error is tolerable given the SNR range used) are effectively best-case scenarios. Thus, these simulations provide best case scenario lower bounds for future empirical work. For example, this approach will not work empirically when co-registration error is greater than a couple of mm, even if everything else is optimal.

We emphasize that there are parameters which we have not fully investigated the effects of. For example, it would be interesting to evaluate the algorithms using different types of correlated noise (although see Fig. 11 in which correlated noise is effectively introduced through multiple sources). Ultimately, there are therefore still unresolved questions related to the assumptions implicit in the algorithms and simulation parameters used here. Given the use of dipolar sources in our set-up, it is thus an empirical question whether the inversion algorithms and generative model will help in reconstructing true hippocampal sources when both spatially distributed simultaneous sources of interference and potentially also more distributed hippocampal activity are present in real data. Nonetheless, we show that irrespectively of these assumptions, source reconstruction of hippocampal activity depends upon accurate co-registration between MRI and MEG data.

Another factor, related to co-registration error, is the accuracy of the parcellation of the hippocampus from the anatomical MRI. In order to simulate errors in this parcellation we added small amounts of shift (Fig. 9 and Supplementary Figure 1), or lateral rotation to the hippocampal structure (Supplementary Figure 3). We find that errors of as little as 0.5 cm or 2 degrees give rise to a significant detriment to the model evidence. One could also see this sensitivity to modelling error as a very positive thing - enabling us to test out new parcellation algorithms (where certain sub-fields are included or excluded for example) and compare them based on model evidence. These rotated hippocampi could also provide an elegant control condition for future empirical studies; for example, one could test if there is more evidence for the aligned versus rotated or shifted hippocampal structure based on the MEG data. Interestingly, compared to the combined models with a straight hippocampus, the rotated hippocampal models are inherently biased to explain more MEG data from artefacts, other brain sources, etc. (see eigenvalue spectra in Supplementary Figure 3) which sets them apart from the cortical models. Therefore, when the correctly aligned hippocampal mesh explains more data it is a yet more compelling demonstration that the source is of hippocampal origin.

### Outlook

The central question of whether significantly higher Free energy for the combined model is specific to hippocampal activity is supported by three lines of converging evidence: *a*) Free energy is not higher for the combined model when the source(s) is/are cortical (Fig. 5**B** and Fig. 11), even when simulating activity on the nearest portion of medial temporal lobe (Fig. 8), *b*) significant Free energy improvement is specific to models with the correct location and orientation of the mesh (Fig. 9, Supplementary Figures 1 and 3), and *c*) the correct source is identified when the correct model is used: the dipole localisation error is ~0 mm at low co-registration error and high SNR (Fig. 10). Thus, the extent to which Free energy differences can be used to infer hippocampal activity is dependent on the accuracy of the solution obtained with the combined model (i.e. when there is too much co-registration error or SNR is too poor, the Bayes Omnibus Risk shows that model differences are unreliable).

We find that the most important empirical factor when attempting to unambiguously determine the presence or absence of hippocampal activation is minimization of co-registration error, and that this is true largely irrespective of noise added at sensor level. Notably, we base the detection of hippocampal on six cycles of oscillatory activity here (20 Hz simulation frequency and 300 ms duration). Having more data would increase the detectability by improving the SNR (Brookes et al., 2008).

The outstanding issue therefore is whether the proposed generative model will be useful in practice. We know from these simulations that the main empirical constraint will be co-registration error which we can now reduce to <1.5 mm using flexible and subject-specific head-casts for MEG (Meyer et al., 2017). Moreover, the head-casts reduce head movement during recording to <0.25 mm which gives way to higher SNR data through repeating larger number of trials with the head location kept constant. We are now working on providing empirical validation of the model comparison approach presented using these devices in conjunction with a paradigm known to modulate hippocampal activity (Doeller et al., 2008).

However, with regards to implementing this scheme in analysis pipelines of real data, a few considerations are worth noting. For example, it is not clear how to most clearly demonstrate that this method works empirically. This is made more challenging by the fact that it is non-trivial to get a good control condition. The question is, when is the hippocampus consistently *not* active? Ideally one would have two task conditions, one designed to preferentially activate the hippocampus and one designed to not do so. However, although the hippocampus may well be more active during one of these phases, it is very unlikely that it will be quiescent during the other. In other words, it may be difficult to show an interaction between task and hippocampal model. That said, one can imagine that with a cognitive task known to engage the hippocampus (for example Doeller et al., 2008), there would be several stages to the analysis. Firstly it would be important to demonstrate that the hippocampal structure is essential to explain the measured data; and then one could look at whether the power in the theta band (for example) within this structure modulates with task demands as observed elsewhere (Bush et al., 2015). Further, it would be interesting to examine whether the hippocampal mesh manipulations shown here reveal selective advantages when inverting using the correctly aligned model.

The roles of the hippocampus in cognition has been emphasized in both humans (for example, Burgess et al., 2002; Lega et al., 2012; Rutishauser et al., 2010; Zhang and Jacobs, 2015), and animals (Kahana et al., 2001, Logothetis et al., 2012). Our work suggests that by using new recording techniques, namely head-casts (Meyer et al., 2017, Troebinger et al., 2014b) we have the ability to selectively study human hippocampal dynamics non-invasively.
