# Supplementary material for: Using generative models to make probabilistic statements about hippocampal engagement in MEG
Source: Neuroimage. 2017 Apr 1;149:468–82. doi: 10.1016/j.neuroimage.2017.01.029 (PMC5387160; doi:10.1016/j.neuroimage.2017.01.029)
Supplement: Supplementary file 1 — Supplementary material [file mmc1.docx]

Supplementary material

*
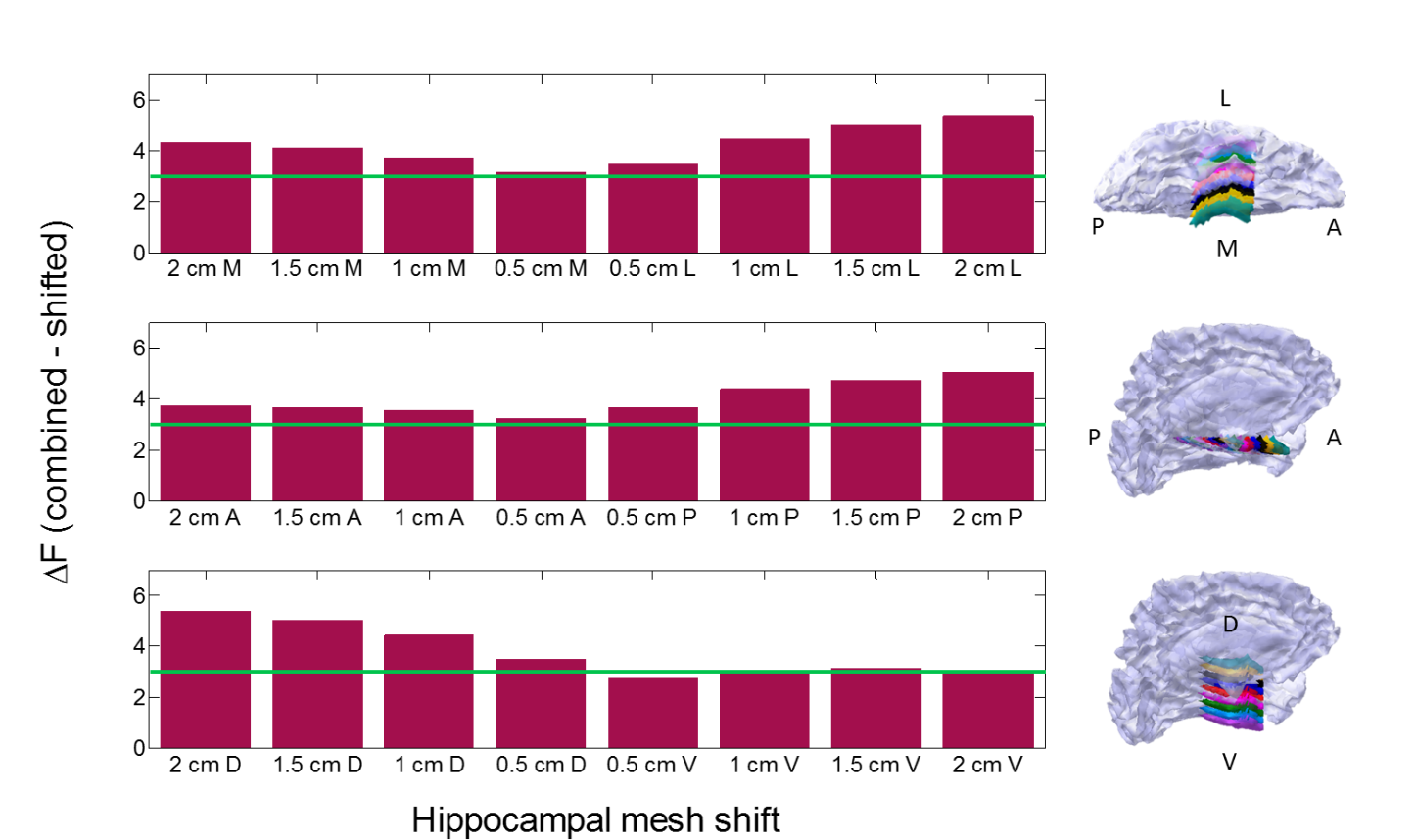
*

**Supplementary Figure 1**: Effect of shifting the hippocampal mesh on Free energy: Comparing models with correct cortical but a shifted hippocampal mesh to the standard combined model (hippocampus in the correct location). Bars represent ΔF = mean F_combined_ – F_shifted_ of 30 different hippocampal simulations using a given shifted model. Top panel shows medial-lateral shifts, middle panel anterior-posterior, bottom panel dorsal-ventral. The correct combined model is significantly better (ΔF>3, green line) than the shifted model. We used SNR -5 dB, no co-registration error, and EBB. L lateral, M medial, P posterior, A anterior, D dorsal, V ventral.


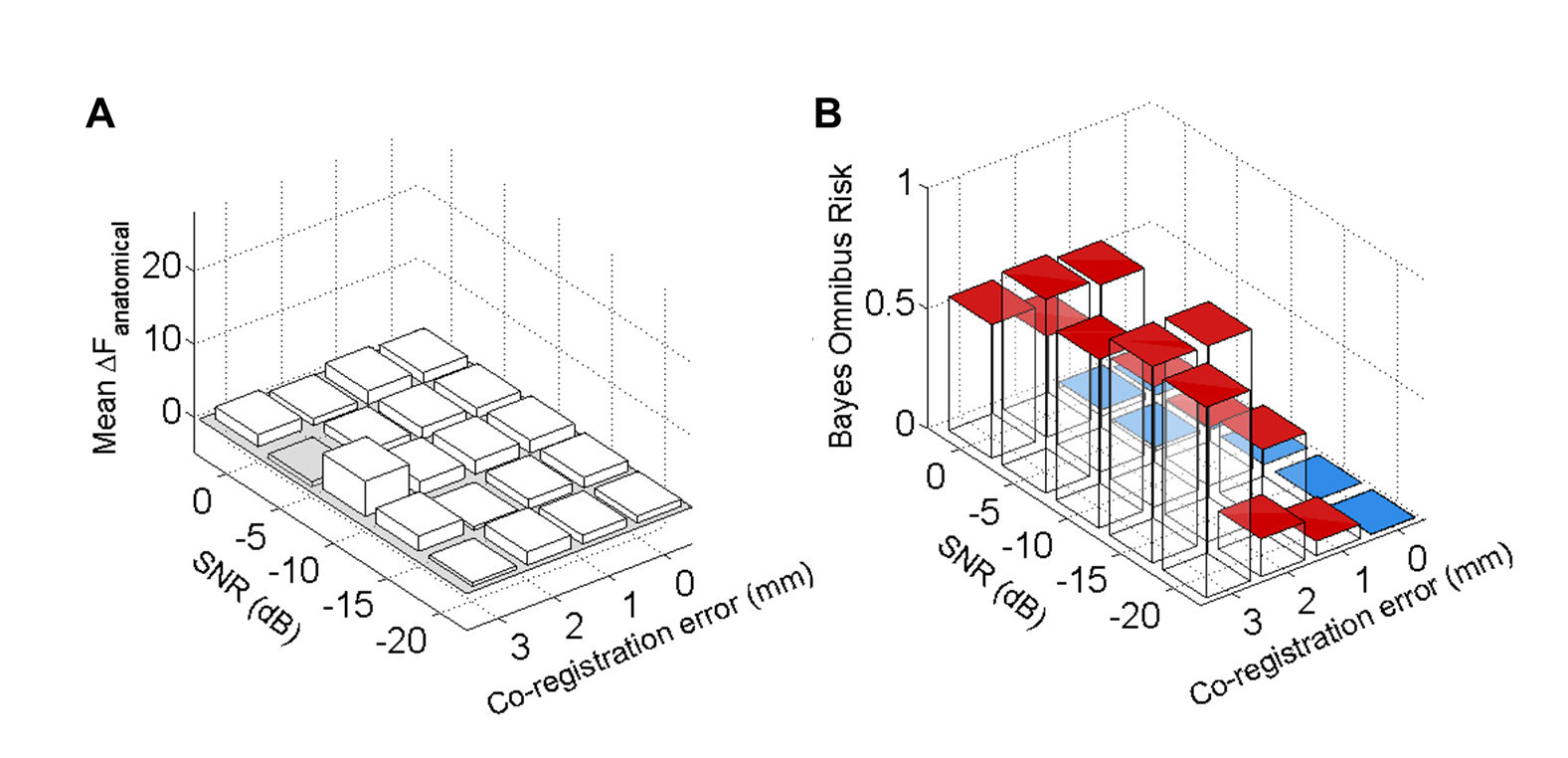


**Supplementary Figure 2**: Effects of noise and co-registration error on anatomical model comparison based on hippocampal simulations using LORETA (Low Resolution Brain Electromagnetic Tomography). **A)** Each bar encodes average ΔF_anatomical_ of 30 reconstructed hippocampal simulations. Z-axis is kept equal to Figure 7 for comparison. Note that the average ΔF for 3 mm of co-registration error and SNR -5 dB is negative, indicating an (incorrect) preference for the cortical model over the combined. **B)** Effects of noise and co-registration error on the Bayesian Omnibus Risk, the risk that anatomical model frequencies are equal. Red bar tops mark BOR values >0.05, blue mark values below. Co-registration errors above 0 mm mean that it is no longer possible to reliably distinguish between models based on F.

**
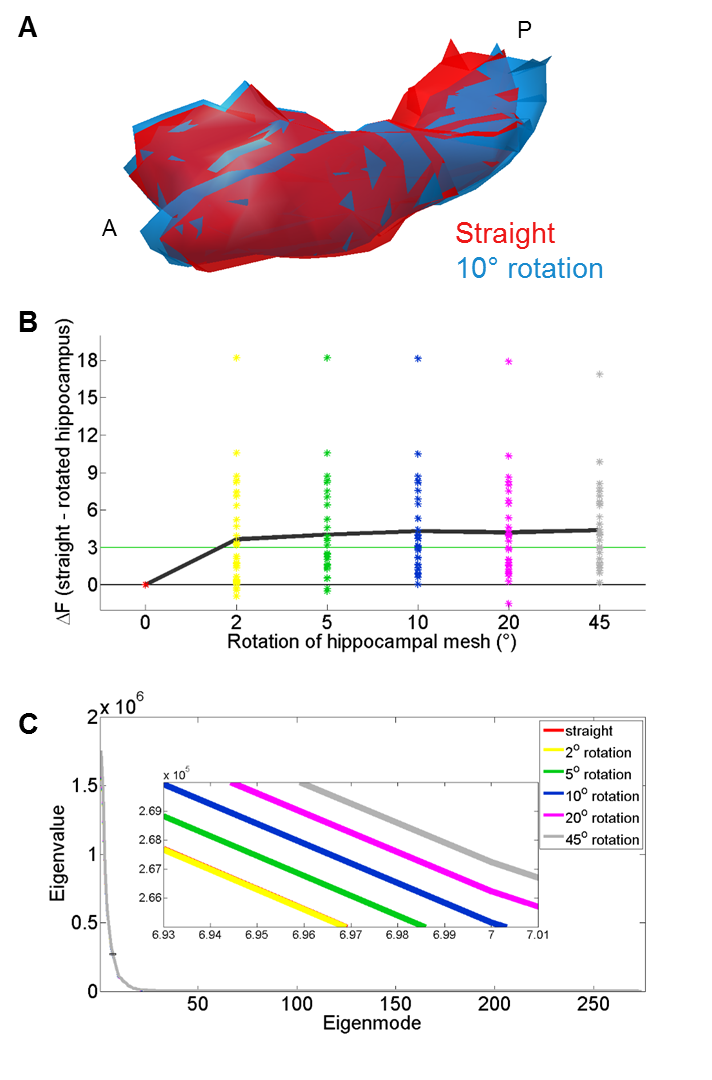
**

**Supplementary Figure 3: A)** Visualisation example showing 10° rotation of hippocampus (blue) relative to straight (non-rotated) hippocampus (red)**.** A anterior, P posterior. **B)** To test whether the model comparison framework is sensitive to errors in parcellation, location or orientation of the hippocampal mesh, we compared F values obtained with the standard combined model to F values obtained with a combined model where a rotation along the longitudinal axis was added to the hippocampus. Coloured asterisks represent 30 ΔF_anatomical_ values obtained with different degrees of hippocampal mesh rotation. Simulations were carried out at SNR = -5 dB and with no co-registration error added. Simulation locations were the same as used previously (drawn at random). As error is added to the hippocampal portion of the generative model, Free energy decreases and ΔF_anatomical_ values significantly (>3) increase on average (thick black line). Even very small rotations (2 degrees) significantly increase ΔF_anatomical._ **C** Comparison of eigenvalues across eigenmodes of leadfields obtained with different models. Inset shows expanded section of plot highlighting difference between models with different degrees of hippocampal mesh rotation (rectangle on main plot marks origin of inset). The more rotation, the higher the eigenvalue for a given eigenmode. In other words, the rotated mesh models (unlike the models in which the hippocampal mesh was excluded) are inherently biased towards explaining more MEG data (from artefacts, other brain sources etc) than the straight mesh models. Thus, if the straight mesh combined model explains more data than the rotated, it is an even more compelling demonstration that the source is of hippocampal origin.


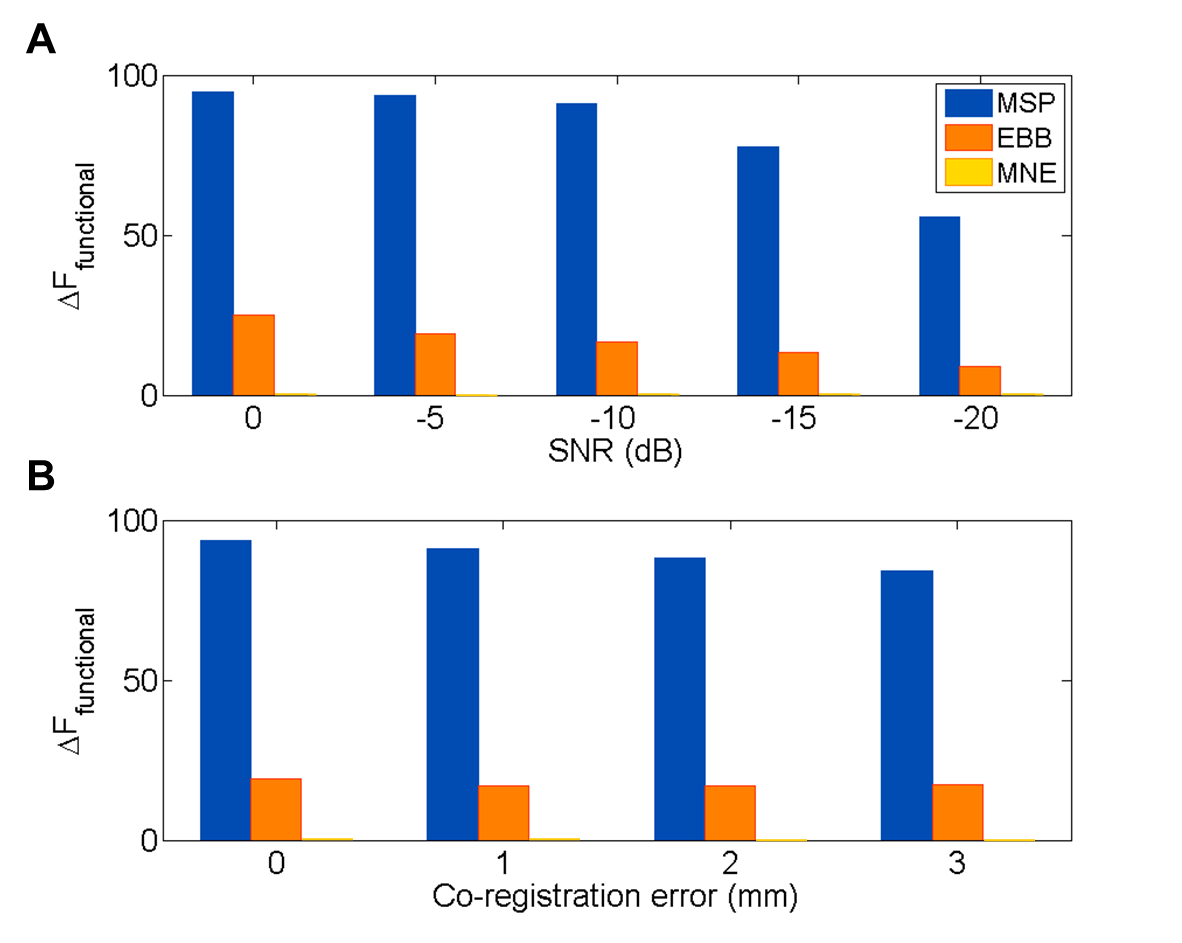


**Supplementary Figure 4:** Functional model comparison. This analysis compares combined models across algorithms, meaning that we compare the functional, as opposed to anatomical, priors. Given that data and models are constant, the differences in Free energy reflect the appropriateness of the assumptions related to source covariance. **A)** Model comparisons of combined models across algorithms as a function of SNR. Bars encode mean functional Free energy difference (F_algorithm_ – F_worst algorithm_) of 30 hippocampal source inversions. Results are normalized to smallest mean functional Free energy difference at each SNR value. Results are for the combined model without co-registration error. The most likely functional prior is MSP for all SNR levels tested, and the second most likely is EBB throughout. This implies that EBB has a relatively good data-driven estimate of source power, even at low SNR (considering that it does not have the explicit advantage of MSP where a small subset of correct and sparse priors are used to constrain the source space). **B)** Same as A, but as a function of co-registration error. Results are similar to SNR range, implying that the model comparison differences are driven by differences in the model evidence values associated with the combined (compared here), and not the cortical models. SNR set to -5 dB throughout.

Appendix A

*Covariance matrix specification using different functional priors*

Bayesian MEG inversion algorithms differ only with respect to definition of the prior source covariance matrix $Q$ (Mosher et al., 2003; Wipf and Nagarajan, 2009). Here we briefly describe the differences between $Qs$ specified using Minimum Norm Estimation (MNE), Empirical Bayes Beamformer (EBB) and Multiple Sparse Priors (MSP).

The standard minimum norm estimation (MNE) represents the simplest assumption about prior source covariance $Q$, namely that the sources are independent and identically distributed. This means that they have the same variance and no covariance and can therefore be described by the covariance matrix:

|  | $Q_{MNE}=I$ | (1) |
| --- | --- | --- |

Where ${I\mathbb{\in R}}^{N_{c}\times N_{c}}$ is an $N_{c}$ sensors by $N_{c}$ sensors identity matrix.

The Empirical Bayes Beamformer (EBB) inversion scheme is similar to MNE as there is a single diagonal source prior covariance matrix which can be written as:

|  | $Q_{EBB}=diag(\sigma^{2})$ | (2) |
| --- | --- | --- |

Where $\sigma^{2}$ is the source variance. Unlike MNE, this variance value is estimated directly from the data under the standard beamforming assumption that there are no spatially separated but temporally correlated sources (Belardinelli et al., 2012; Van Veen et al., 1997). Thus, while MNE assumes that all sources are potentially active with equal probability, EBB selects and weights priors based on their putative contribution to the measured signal. Specifically, for every dipolar location θ, the source variance $\sigma^{2}$ is calculated as follows (Hillebrand and Barnes, 2005; Mosher et al., 2003):

|  | $\sigma_{\theta}^{2}={(L_{\theta}^{T}C_{b}^{-1}L_{\theta})}^{-1}$ | (3) |
| --- | --- | --- |

Where$C_{b}=YY^{T}$ is the sensor-level covariance matrix and $L_{\theta}$represents the effective, smoothed lead field for a patch centred at dipole location θ. (·)^T^ denotes a transpose operator.

The Multiple Sparse Priors (MSP) inversion (Friston et al., 2008a) models a set of sparse local spatial patches (as opposed to a single cortex-wide pattern) and prunes away those patches which do not explain variance. Importantly, MSP is a more general form of the approaches described above because the structure of the prior components in $Q$ can take any other form (if it is more appropriate), including those of EBB and MNE. This is because the prior source covariance is a weighted sum of a set of (multiple sparse) prior components, one per spatial prior: $Q=\{C_{1},\ldots,C_{N_{q}}\}$ where $N_{q}$ denotes the number of priors covering the mesh. The default $N_{q}=512$ in SPM but in our case we use only $N_{q}=$ 100 randomly spaced spatial priors (patches) for computational simplicity. The priors constrain the source space such that the algorithm is forced to explain the data using the priors given such that a poor model (i.e. a set of incorrect priors) will have a low model evidence. Both generative models used with MSP have 90 identical randomly distributed cortical priors but differ on the inclusion of hippocampal priors (the remaining 10 cortical priors in the cortical model are also randomly distributed across the cortex). The (global) prior source covariance matrix can be expressed as:

|  | $Q_{MSP}= \sum_{i=1}^{N_{q}} h_{i}C_{i}$ | (4) |
| --- | --- | --- |

Where each ${C_{i}\mathbb{\in R}}^{N_{d}\times N_{d}}$ is itself a prior source covariance matrix with each prior component corresponding to a smooth surface patch. These covariance components are individually weighted by hyperparameters $h$ = {$h_{1}$, …, $h_{N_{q}}$}. The larger a given hyperparameter, the larger the prior variance of the patch. MSP can thereby optimize the hyperparameters so as to best fit the modelled covariance to the data covariance (at sensor-level) by mixing and pruning (hyperparameter down-weighting) the priors such that the model evidence is maximized. Whereas MNE and EBB use only a single hyperparameter to fit the data covariance, MSP uses one per spatial prior.

After implementing the functional prior assumptions as described above, the algorithm-specific $Q$is empirically optimised whereby that the current density can be estimated and most likely source distribution inferred. The optimisation is based on an approximate Bayesian inversion scheme, Variational Laplace (Friston et al., 2008b), which assumes that the posterior distribution of $J$ (${J\mathbb{\in R}}^{N_{d}\times N_{t}}$ which describes the amplitude of $N_{d}$ current dipoles over $N_{t}$ time samples) is Gaussian. The result is a set of hyperparameters that maximize the model evidence for the given data, and which are used to specify $Q$ in the subsequent data inversion step (see for example (Grech et al., 2008) for details).

Appendix B

*Code for simulating and reconstructing hippocampal activity is submitted alongside the manuscript.*

*The link for downloading the annotated code and data files is:*

<https://github.com/sofiemeyer/Meyeretal_NeuroImage_2017_SimulationScript>

*This code interfaces with the SPM12 Matlab Toolbox.*
